# Pregnancy Prevention and Condom Use Practices among HIV-Infected Women on Antiretroviral Therapy Seeking Family Planning in Lilongwe, Malawi

**DOI:** 10.1371/journal.pone.0121039

**Published:** 2015-03-26

**Authors:** Lisa B. Haddad, Caryl Feldacker, Denise J. Jamieson, Hannock Tweya, Carrie Cwiak, Thomas Chaweza, Linly Mlundira, Jane Chiwoko, Bernadette Samala, Fanny Kachale, Amy G. Bryant, Mina C. Hosseinipour, Gretchen S. Stuart, Irving Hoffman, Sam Phiri

**Affiliations:** 1 Emory University School of Medicine, Department of Gynecology and Obstetrics, Atlanta, Georgia, United States of America; 2 The Lighthouse Trust, Lilongwe, Malawi; 3 International Training and Education Center for Health (I-TECH), University of Washington, Seattle, Washington, United States of America; 4 University of North Carolina Chapel Hill, Chapel Hill, North Carolina, United States of America; 5 University of North Carolina Project-Malawi, Lilongwe, Malawi; 6 Reproductive Health Services, Ministry of Health, Lilongwe, Malawi; FIOCRUZ, BRAZIL

## Abstract

**Background:**

Programs for integration of family planning into HIV care must recognize current practices and desires among clients to appropriately target and tailor interventions. We sought to evaluate fertility intentions, unintended pregnancy, contraceptive and condom use among a cohort of HIV-infected women seeking family planning services within an antiretroviral therapy (ART) clinic.

**Methods:**

200 women completed an interviewer-administered questionnaire during enrollment into a prospective contraceptive study at the Lighthouse Clinic, an HIV/ART clinic in Lilongwe, Malawi, between August and December 2010.

**Results:**

Most women (95%) did not desire future pregnancy. Prior reported unintended pregnancy rates were high (69% unplanned and 61% unhappy with timing of last pregnancy). Condom use was inconsistent, even among couples with discordant HIV status, with lack of use often attributed to partner’s refusal. Higher education, older age, lower parity and having an HIV negative partner were factors associated with consistent condom usage.

**Discussion:**

High rates of unintended pregnancy among these women underscore the need for integ rating family planning, sexually transmitted infection (STI) prevention, and HIV services. Contraceptive access and use, including condoms, must be improved with specific efforts to enlist partner support. Messages regarding the importance of condom usage in conjunction with more effective modern contraceptive methods for both infection and pregnancy prevention must continue to be reinforced over the course of ongoing ART treatment.

## Introduction

Family planning is a cost effective intervention for preventing both maternal-to-child transmission (PMTCT) of human immunodeficiency virus (HIV) and maternal morbidity and mortality from unintended pregnancy [1–4]. In areas such as sub-Saharan Africa, where HIV is endemic among heterosexual populations, target audiences for HIV and family planning (FP) services overlap and can benefit from integrated services [5–10]. In recognition of goals to prevent pregnancy, transmission of HIV and other sexually transmitted infections, FP programs operating in HIV care settings must promote dual methods: condoms to prevent infection transmission and another more effective contraceptive for pregnancy prevention [11].

Despite evidence that dual protection with condoms and a concurrent, more effective, contraceptive method would be advantageous, barriers to family planning and condom use remain. Well known barriers to family planning include lack of female decision-making power, poor economic resources, low quality care at family planning services, and desire for large families [12, 13]. Given the importance of dual method use, perceptions and use of contraceptives, including condoms, among HIV-positive women merit special attention. Understanding factors that influence practices among HIV+ women already on antiretroviral therapy (ART) is critical as access to ART increases. However, these issues currently remain poorly understood among this specific population of women.

Several studies have explored the impact of HIV infection on women’s fertility decisions and pregnancy rates [14–20]. Prior studies found that HIV-infected women have a decreased desire for children in comparison to their uninfected peers[11, 21]; however, this reduced desire seems to be diminishing in regions with improved overall health status for HIV-infected women[20, 22]. Evidence suggests that socio-cultural factors play a large role in fertility decision-making and that there is a rich and complex range of factors, including HIV status and ART use, which influence reproductive decisions [20, 23]. Given the importance of dual method use, the expansion of ART across the region, and increasing interest in the role of ART as a preventive method in reducing HIV infectivity, information on sexual practices among HIV+ women on ART is critical. Through increased understanding of this population’s needs, we can target our interventions to address the challenges and obstacles that reduce safe sexual practices.

Therefore, we investigated contraceptive practices as well as unintended pregnancy and condom usage among a cohort of 200 HIV-infected women receiving ART who presented for family planning services at a public ART clinic in Lilongwe, Malawi. Further, we explored factors associated with condom usage, as use is an essential independent component of HIV and sexually transmitted infection prevention strategy. By exploring these factors, we hope to inform appropriate and effective FP/ART integration efforts at the programming and policy levels.

## Methods

This analysis used cross sectional data obtained at baseline from 200 women who consented to enroll in a prospective randomized contraceptive trial comparing the copper T380A intrauterine device (IUD) to depot medroxyprogesterone acetate (DMPA); the study design and population was described previously [24, 25]. Briefly, the study population consisted of HIV-infected women in Lilongwe, Malawi who attended the Lighthouse clinic at Kamuzu Central Hospital (KCH) and desired family planning. The Lighthouse is a Centre of Excellence for integrated HIV Management and operates two clinics, one at KCH and another based at Bwaila Maternity Hospital in Lilongwe, Malawi. The Lighthouse Clinic at KCH, at which this study was conducted, has over 20,000 patients on ART and over 2,000 patients who are not yet clinically eligible for ART.

To be eligible for the study women were between 18–45 years of age, HIV+ and on ART for at least 6 months, desired to avoid pregnancy for at least 12 months, and had no contraindications to DMPA or the copper IUD [26]. A total of 281 women were screened between August 2010 and December 2010 to enroll 200 women willing to be randomized to receive either DMPA or the IUD. There was no difference between mean age, gravidity, or parity between enrolled women and those not enrolled in the study [26]. The baseline questionnaire administered to the 200 women enrolled was used for this current analysis.

This baseline questionnaire included information on: demographics, medical history, fertility intentions, sexual behaviors, contraceptive beliefs and preferences, and condom use. Questions were a compilation of original study questions and questions used in the Malawi 2008 Demographic and Health Survey (DHS) [27]. Questions were pilot tested prior to the study and administered in Chichewa by an interviewer using a paper-based, semi-structured questionnaire.

A study database in Microsoft Access 2003 (Microsoft, Redmond, WA, USA) was created for the data entry and management. All data were entered using double entry and validated using predetermined queries. SPSS version 17.0 (SPSS Inc, Chicago, IL, USA) was used for the statistical analysis. Descriptive statistics were used to report patterns of family planning, fertility intentions, unplanned pregnancy, and self-reported condom use. As condoms were the most commonly reported method of contraception used among this cohort, bivariate and multivariate logistic regression was used to determine odds ratios for potential factors associated with condom utilization. Categorical variables were not included in this analysis if >95% of the cohort fell into any one of the categories. Any current or past contraceptive use was included in the analysis; however, any association with a specific method used was not evaluated. Condom use was evaluated using two metrics: self-reported condom usage during last coitus or consistent condom usage (defined as self-report of always using a condom during intercourse in the past 12 months). Multivariate logistic regression models contained all variables from the bivariate analyses where the 95% confidence interval of the unadjusted odds ratio for at least one comparison group within each categorical variable did not include 1. Associations are reported as odds ratios (ORs) with 95% confidence intervals (CIs). Potential factors of interest in the model included: age, education, marital status (married or unmarried), current relationship status (monogamous, non-monogamous, unknown, no current relationship), length of time on ART (less than 24 months or 24 months or longer), gravidity (2 or fewer prior pregnancies or more than 2 prior pregnancies, abortion history (none or 1 or more prior spontaneous or induced), HIV status of partner, history of sexually transmitted infections, previous modern contraceptive use (including any hormonal contraceptive or IUD), current modern contraceptive use, desire for more children, perceived partner’s desire for more children, and perceived partner support of current use of birth control. For continuous measures such as ART duration and Gravidity for which we created categorical variables, we examined the data using descriptive statistics and consulted the literature, creating categories based on median values, natural breaks, and common categories from similar studies.

We received support and formal approval to conduct the study in Malawi from all sites involved including the Ministry of Health, National Health Services Research Committee in Malawi (protocol #717), the institution review board at Emory University (IRB00037535), and the institutional review board at University of North Carolina-Chapel Hill (study #10-1352). We have written informed consent from all participants prior to participation. The primary study from which this data is abstracted is posted on ClinicalTrials.gov (identifier: NCT01191203).

## Results

Most women were between 26 and 35 years of age, married and poorly educated, with 51% of the women reported having 4 or more prior pregnancies and most reporting no prior abortions (Table 1). Most of the women (90%) had one partner in the past year, reported being in a current relationship (88.5%), and lived with their partner (78%). Approximately half reported mutual monogamy with their partner (54.5%). About 70% of the women were aware of their partner’s HIV status while almost 30% (29.4%) did not know their partner’s status. Most women stated that their partner was aware of their HIV-positive status.

**Table 1:** Characteristics of 200 women enrolled in study.

	n = 200	%
Age mean (SD)	32.3 (5.6)	
< = 25	20	10.0
26–35 years old	126	63.0
>36	54	27.0
Educational status		
None or some primary school	94	47.2
Completed primary school or more	105	52.8
Missing	1	
Marital status		
Single	8	4.0
Married monogamous	128	64.0
Married polygamous	15	7.5
Divorced/widowed	31	15.5
Do not know/decline	18	9.0
Relationship status		
Mutually monogamous	109	54.5
Not monogamous	26	13.0
Unsure if monogamous	42	21.0
No current partner	23	11.5
Number of pregnancies		
None	3	1.5
One	14	7.0
Two	31	15.5
Three	50	25.0
Four or more	92	46.0
History of an abortion		
No	145	72.5
Yes	55	27.5
Number of partners in past year		
None	9	4.5
One	180	90.0
Two or more	11	5.5
Is your partner HIV+		
No	19	10.7
Yes	106	59.9
Unsure of status	52	29.4
N/A	23	
Does your partner know you are HIV+		
No	3	1.7
Yes	170	96.0
Unsure	4	2.3
N/A	23	
Length of time on ART		
< = 24 months	104	52.0
>24 months	96	48.0
History of any STI other than HIV		
No	159	79.5
Yes	41	20.5

N/A = not applicable, ART = Antiretroviral therapy, STI = Sexually transmitted infection.

The majority of women reported a prior unintended pregnancy (Table 2): 69.9% reported that their last pregnancy was unplanned and 61% were unhappy with the timing of that pregnancy. Only 31 (15.7%) women reported using birth control at the time of their last conception: among these women approximately half (45.2%) reported condom use and the other half reporting either DMPA (38.7%) or oral contraceptive pill usage (16.1%). Most women stated that neither they nor their partner desired more children in the future and that their partner wanted them to use a contraceptive method. Despite the high desire to limit future fertility, only 10 women reported current use of a family planning method other than condoms. Although current contraceptive use other than condoms was low, many women (69.5%) had used contraception in the past.

**Table 2:** Fertility intentions, unplanned pregnancy, contraception and condom use among HIV+ clients enrolled in study.

	n = 200	%
Last pregnancy		
Planned	59	30.1
Unplanned	137	69.9
Missing	1	
N/A	3	
Happy with timing of last pregnancy		
Yes, happy with timing	75	37.5
No, unhappy with timing	122	61.0
Missing/NA	3	
Using birth control when most recently pregnant		
No	166	84.3
Yes	31	15.7
N/A	3	
Method used when got pregnant		
OCP	5	16.1
DMPA	12	38.7
Male condoms	14	45.2
N/A	169	
Do you want more children in the future		
No	190	95.0
Yes	10	5.0
Partner wants more children		
No	146	82.5
Yes	13	7.3
Do not know	18	10.2
N/A	23	
Partner desires family planning use		
No	9	5.1
Yes	151	85.3
Do not know	17	9.6
N/A	23	
Any modern contraceptive method ever used other than condoms		
No	61	30.5
Yes	139	69.5
Contraceptive methods previously ever used		
OCP	39	19.5
Implant	5	2.5
Male Condom	184	92.0
Female Condom	17	8.5
DMPA	128	64
IUD	5	2.5
Emergency contraception	0	0
Rhythm method	4	2
Withdrawal	2	1
Abstinence	27	13.5
Contraceptive method currently using		
None	53	26.6
OCP	1	0.5
DMPA	9	4.5
Male Condom	131	65.8
Female Condom	1	0.5
Rhythm method	1	0.5
Abstinence	3	1.5
Missing	1	
Condom use in past 12 months		
Never	17	8.6
Sometime	110	55.6
Always	70	35.9
Condom use at last coitus		
No	75	37.9
Yes	123	62.1

OCP = Oral contraceptive Use, DMPA = Depot medroxyprogesterone acetate, IUD = Intrauterine device, N/A = not applicable.

In evaluating condom use, 131 women (65.8%) reported current use. However consistent condom usage, defined as always using a condom in the past 12 months, was reported by only 35.9% (n = 71) of the women. More women reported using condoms some of the time (n = 110, 55.6%), and more than half the women (n = 123, 62.1%) did use a condom at last coitus.

127 women (64.5%) reported inconsistent condom use, with many factors being responsible. The most common reason for reported non-use was their partner (n = 95, 77.2%), with the partner having difficulty (n = 63, 51.2%), discomfort (n = 20, 16.3%), objecting (n = 7, 5.7%), or being drunk (n = 5, 4.1%) as the reasons cited. Lack of availability was another common reason for non-use reported by 15 women (12.2%). Factors related to the woman (e.g. female discomfort) or related to the couple (e.g. trusting each other) were uncommon reasons for condom non-use, each reported by only 3 women (2.4%).

When we evaluated factors associated with consistent condom use (Table 3), we found women younger than 25 years (adjusted odds ratio (aOR) 0.33, 95% CI 0.13–0.85) with 3 or more prior pregnancies (aOR 0.38, 95% CI 0.21, 0.68) or those reporting a history of an abortion (aOR 0.36, 95% CI 0.20–0.65) were less likely to use condoms consistently, while women with higher education (completion of secondary school or more) (aOR 3.85, 95% CI 2.27–6.51) were more likely to employ consistent condom usage. Having an HIV-negative partner was associated with an approximately 5 times increased odds of consistently using condoms compared to those with HIV-positive partners (aOR 0.22, 95% CI 0.10–0.52) or those who did not know their partner’s status (aOR 0.20, 95% CI 0.08–0.52). Although women in discordant HIV status relationships were more likely to use condoms, 31.6% of these couples did not use a condom consistently (data not shown). Lastly, women with partners who do not support family planning use were significantly more likely to use condoms consistently compared to those whose partners supported family planning or who did not know if their partner supported family planning.

**Table 3:** Factors associated with consistent condoms in last year and condom usage at last intercourse, unadjusted and adjusted odds ratios with significant variables from bivariate evaluation included in the model.

	****Consistent Condom Usage****		****Condom Usage at Last Coitus****	
Factor	UNADJUSTED OR (95% CI)	ADJUSTED OR (95% CI)	UNADJUSTED OR (95% CI)	ADJUSTED OR (95% CI)
Age				
<25	0.42 (0.18, 0.94)	0.33 (0.13, 0.85)*	0.34 (0.13, 0.89)*	0.24 (0.08, 0.73)*
25–34	1 (Ref)	1 (Ref)	1 (Ref)	1 (Ref)
>35	1.01 (0.63, 1.61)	1.30 (0.73, 2.33)	0.77 (0.40, 1.50)	0.97 (0.445, 2.113)
Education				
None or some primary school	1 (Ref)	1 (Ref)	1 (Ref)	1 (Ref)
Completion of primary school or more	2.61 (1.42, 4.80)*	3.85 (2.27, 6.51)*	2.66 (1.46, 4.81)*	4.60 (2.17, 9.76)*
Marital status				
Single/Divorced/Widowed	1 (Ref)		1(Ref)	
Married	1.24 (0.58, 2.66)		1.62 (0.79, 3.35)	
Relationship status				
Monogamous	1 (Ref)	1 (Ref)	1 (Ref)	1 (Ref)
Non-monogamous	1.03 (0.55, 1.95)	0.76 (0.34, 1.73)	0.32 (0.13, 0.77)*	0.18 (0.06 0.56)*
Do not know	2.14 (1.28, 3.57)*	1.92 (1.02, 3.61)*	0.71 (0.34, 1.49)	0.54 (0.22, 1.35)
No current partner	0.32 (0.13, 0.81)*	0.00 (0.00, 0.02)*	0.40 (0.15, 1.02)	0.01 (0.00, 0.20)*
Number of partners in last year				
0	n/a		1 (Ref)	
1	1 (Ref)		3.07 (0.71, 13.27)	
> = 2	0.36 (0.08, 1.70)		0.95 (0.14, 6.28)	
ART duration				
0–24 months	1 (Ref)		1 (Ref)	
>24 months	0.71 (0.39, 1.28)		0.75 (0.41, 1.36)	
History of STI				
No	1 (Ref)		1 (Ref)	
Yes	0.59 (0.28, 1.27)		0.64 (0.32, 1.29)	
Prior contraceptive use^1^				
No	1 (Ref)		1 (Ref)	
Yes	1.22 (0.64, 2.30)		1.26 (0.68, 2.34)	
Current contraceptive use^2^				
No	1 (Ref)		1 (Ref)	
Yes	1.88 (0.52, 6.72)		1.46 (0.37, 5.83)	
Pregnancy number				
0–2	1 (Ref)	1 (Ref)	1 (Ref)	1 (Ref)
3 or more	0.43 (0.22, 0.84)*	0.38 (0.21, 0.68)*	0.40 (0.19, 0.84)*	0.39 (0.16, 0.97)*
History of an abortion				
No	1 (Ref)	1 (Ref)	1 (Ref)	1 (Ref)
Yes	0.45 (0.22, 0.92)*	0.36 (0.20, 0.65)*	0.48 (0.25, 0.90)*	0.46 (0.21, 0.97)*
Partner’s HIV status				
Negative	1 (Ref)	1 (Ref)	1 (Ref)	
Positive	0.26 (0.09, 0.73)*	0.22 (0.10, 0.52)*	0.79 (0.26, 2.38)	
Do not know	0.22 (0.07, 0.69)*	0.20 (0.08, 0.52)*	0.36 (0.11, 1.14)	
Desire more children				
No	1 (Ref)		1 (Ref)	
Yes	2.84 (0.77, 10.42)		1.45 (0.36, 5.78)	
Last pregnancy				
Planned				
No	1 (Ref)		1 (Ref)	
Yes	1.27 (0.68, 2.42)		1.02 (0.54, 1.92)	
Happy with the timing of last pregnancy				
No	1 (Ref)		1 (Ref)	
Yes	1.0 (0.99, 1.00)		1.0 (0.98, 1.00)	
Using Birth control at last pregnancy				
No	1 (Ref)		1 (Ref)	
Yes	1.07 (0.48, 2.40)		1.34 (0.59, 3.04)	
Partner desires more children				
No	1 (Ref)		1 (Ref)	
Yes	2.65 (0.83, 8.50)		1.17 (0.34, 4.00)	
Do not know	0.64 (0.22, 1.88)		0.42 (0.16, 1.12)	
Partner supports FP use				
No	1 (Ref)	1 (Ref)	1 (Ref)	1
Yes	0.07 (0.01, 0.32)*	0.04 (0.01, 0.20)*	0.24 (0.03, 1.96)	0.07 (0.01, 0.73)*
Do not know	0.05 (0.01, 0.27)*	0.02 (0.00, 0.15)*	0.07 (0.01, 0.68)*	0.02 (0.00, 0.31)*

*Statistically significant.

^1^history of use of hormonal contraceptives or IUD.

^2^current use of hormonal contraceptives or IUD.

OR = Odds Ratio, FP = Family planning, ART = Antiretroviral therapy, STI = Sexually transmitted infection.

Similarly, we examined factors associated with condom use at last coitus. As with consistent condom usage, younger age, history of three or more deliveries, and a prior abortion were associated with reduced odds of using a condom, while higher education remained strongly associated with condom use. More women in a monogamous relationship reported using a condom at last coitus, both compared to those with no partner (aOR 0.01, 95% CI 0.0–0.2) and those in non-monogamous relationships (aOR 0.18, 95% CI 0.06–0.56). HIV status of the partner was not significantly associated with condom usage at last coitus. Marital status, a woman’s fertility intention, perceived partner’s desire for more children, current or prior contraceptive use or length of time using antiretroviral therapy was not associated with consistent condom use or use at last coitus.

## Discussion

Among these HIV-positive women in Malawi receiving antiretroviral therapy and seeking family planning services, most did not desire future fertility. Similarly, most women believed that their partners did not desire more children. Despite this, we found inadequate contraceptive use with high rates of unintended and mistimed pregnancies. Unmet contraceptive need in sub-Saharan Africa remains high, surpassing 30% in some countries, and is associated most often with poor access and lack of education [28]. The high proportion of women who get pregnant while using contraception may reflect inconsistent condom usage or poor patient adherence to hormonal contraceptive methods. This is consistent with reports where approximately one-third of unintended pregnancies occur among women who are accessing often less effective, short-term contraception methods that require user adherence on a daily or monthly basis [28, 29] and are associated with high rates of discontinuation [28].

The findings on poor condom use is not surprising given other studies which document ineffective condom usage [30], however it is particularly concerning among this population of HIV-infected women. Condom promotion and use is a critical component of HIV prevention programming[31], yet these high-risk couples, despite patient education, do not consistently use condoms. In part, low condom use may be influenced by the fact that condoms require male participation and acceptance, a factor that may lead women to use other types of contraceptive’s methods without male partner knowledge[12]. In our study, the most common reason reported for condom non-use was related to the male partner, reaffirming that relationship dynamics play a critical role in condom use[32]. Although social acceptability bias in studies also may make it easier for women to attribute non-use to their partner, it warrants additional evaluation of perceived barriers and challenges to condom use among men in concordant positive and in discordant relationships. Further, this highlights the importance of developing additional HIV prevention strategies, including more female controlled modalities, at the macro level that can be used without partner knowledge or approval. In the immediate term, improved counseling on family planning for men and women, in conjunction with promotion of couples counseling for family planning, may be an important approach at the clinic that may improve both condom adherence and family planning utilization.

Factors associated with inconsistent condom use, such as younger age, lower education, higher number of pregnancies, and history of abortion may reflect both the consequences of condom non-use as well as underlying factors that may limit female empowerment. These factors are similar to those identified in prior sub-Saharan African studies where risk of unintended pregnancy was associated with prior unintended pregnancy, higher number of prior pregnancies, partner’s desire for more children, poor family wealth status, and residing at further distances from health facilities [33–35]. A recent study of pregnancy incidence among women in sero-discordant couples in seven eastern and southern African countries found that unintended pregnancy among contraceptive users was associated with younger age, having at least two children, higher coital frequency, and unprotected sex. [36].

We did not specify abortions as either spontaneous or induced, however even when controlling for number of pregnancies, the number of abortions remained a significant predictor of non-condom use, suggesting that having reported an abortion identifies a high-risk group that needs to be targeted for efforts to improve condom utilization. As abortion is illegal in Malawi, it is also important to explore this finding further given current abortion practices, which may be clandestine or unsafe, creating additional harm potential for these women.

One unexpected finding was that when a partner supports the use of family planning, condoms are less likely to be used consistently. Although prior studies have noted that among HIV-infected women, discussions of family planning with a partner increased the likelihood of contraceptive use [37–39], others have shown that among use of modern contraceptive methods can lead to a decrease in condom use[40–42]. Similarly in our population, the partners who support family planning may not support dual method use with condoms when the woman uses a different method. Additionally we found that duration of ART use was not associated with condom usage. Although it is possible that women on ART may reduce their concern about HIV transmission concurrent with their drop in viral load, this does not appear to be an issue in our population. Given the overall poor condom usage among this cohort, however, ongoing education is imperative and further evaluation of these trends is warranted in the setting of increased focus on ART as an HIV preventive strategy. Additionally, other studies have suggested that among HIV-infected women, contraceptive use might also change over time on ART, [43, 44] possibly as women return to health, changing desires for family, or due to concerns about interactions of contraceptives and ART [12]. This trend in the literature warrants further evaluation.

A limitation of our findings is that we rely on subjective retrospective reporting. Retrospective reporting is influenced by recall bias and therefore may not be accurate. For example, we used a retrospective measure to define unintended pregnancy. This may reflect a post-birth rationalization bias which could lead to underestimates of unintended pregnancy [45]. Similarly, reported partner’s fertility intentions, knowledge of HIV status, and support of family planning is limited to the woman’s perception of her partner, which may be shaped by cultural expectations, partner communication and relationship factors. Non-inclusion of the partner in this assessment may have allowed women to externally attribute behavior related to inconsistent condom use to their partners. Also, as either men and women may bring HIV into a marriage, it is critical that, future research efforts also include partner’s condom perceptions, attitudes, and practices to better inform family planning policy and practice [46, 47]. Lastly, although a strength in our evaluation is that we evaluate two different measures of condom use, we acknowledge that self reported condom usage has been shown in some studies to be an unreliable indicator for true condom usage [48–50]. Future studies should aim to supplement self-reported condom use with biomarkers, such as prostate specific antigen (PSA), as objective measures for semen exposure.

A strength of this study is that we have identified a high-risk group of women who are seeking family planning and are reflective of a population of women who will likely be targeted by contraceptive programs in sub-Saharan Africa. With that said, these results presented are specific to this community of HIV-infected women on ART who were seeking family planning, most of whom did not desire future fertility and were motivated and willing to be randomized in a contraceptive trial. The practices and experiences of these women may be dissimilar to non HIV-infected women, HIV-infected women not on ART or seeking care in another clinic or those who are not amenable to receiving an IUD. Compared to the DHS data in Malawi from 2010[51], the women in our study have had more prior birth control experience and used condoms for birth control more frequently than among all women and among married women. For example, 93.5% of our cohort reported using family planning at some point, compared to 65.8% of all women and 78.7% of married women in the DHS. Further the male condom was reported as being used at some point by 92% of our cohort compared to 18.6% and 19.6% of all women and married women surveyed in the DHS, findings echoed by another study on discordant couples who found that LARC users reported more dual protection than couples using only condoms[52]. Although this highlights that condoms are more familiar and more widely used among this HIV cohort, condoms were still not used consistently and unintended pregnancy rates were high. There is still a need for promotion of alternative, more effective contraceptive strategies in addition to condoms among HIV-infected women, even among those who are motivated to use alternative family planning methods and whom report prior condom use.

## Conclusion

As most family planning methods are safe in this population and unintended pregnancy is an ongoing concern, our results highlight that efforts must be made to increase access to and uptake of effective contraceptive options in addition to condoms. Expanding on prior and current programming in the region, we must continue efforts toward integration of family planning into HIV care, provide consistent messaging regarding continued condom use among HIV infected women in addition to antiretroviral adherence, and promote involvement of partners in family planning counseling. As partner relationships often pose a challenge for condom use, efforts need to address these obstacles and promote safe sexual practices at Lighthouse clinic and in similar settings in the region.
